# Population structure, gene flow, and sex‐biased dispersal in the reticulated flatwoods salamander (*Ambystoma bishopi*): Implications for translocations

**DOI:** 10.1111/eva.13287

**Published:** 2021-08-27

**Authors:** Steven T. Williams, Jean P. Elbers, Sabrina S. Taylor

**Affiliations:** ^1^ School of Renewable Natural Resources Louisiana State University AgCenter Baton Rouge Louisiana USA; ^2^ University of Veterinary Medicine Vienna Vienna Austria

**Keywords:** *Ambystoma bishopi*, male‐biased dispersal, migration, population structure, reintroductions, reticulated flatwoods salamander, translocations

## Abstract

Understanding patterns of gene flow and population structure is vital for managing threatened and endangered species. The reticulated flatwoods salamander (*Ambystoma bishopi*) is an endangered species with a fragmented range; therefore, assessing connectivity and genetic population structure can inform future conservation. Samples collected from breeding sites (*n* = 5) were used to calculate structure and gene flow using three marker types: single nucleotide polymorphisms isolated from potential immune genes (SNPs), nuclear data from the major histocompatibility complex (MHC), and the mitochondrial control region. At a broad geographical scale, nuclear data (SNP and MHC) supported gene flow and little structure (*F*_ST_ = 0.00–0.09) while mitochondrial structure was high (Φ_ST_ = 0.15–0.36) and gene flow was low. Mitochondrial markers also exhibited isolation by distance (IBD) between sites (*p* = 0.01) and within one site (*p* = 0.04) while nuclear markers did not show IBD between or within sites (*p* = 0.17 and *p* = 0.66). Due to the discordant results between nuclear and mitochondrial markers, our results suggest male‐biased dispersal. Overall, salamander populations showed little genetic differentiation and structure with some gene flow, at least historically, among sampling sites. Given historic gene flow and a lack of population structure, carefully considered reintroductions could begin to expand the limited range of this salamander to ensure its long‐term resilience.

## 1 INTRODUCTION

Amphibian populations have experienced severe declines worldwide, with up to one third of amphibian species currently facing extinction (McCallum, 2007; O’Donnell et al., 2017). Drivers of this decline include pollution, climate change, exposure to novel diseases, and habitat loss or fragmentation (Grant et al., 2016; McCallum, 2007). Increased habitat loss and fragmentation have reduced population sizes and inhibited gene flow among amphibian populations, gene flow that may prevent extirpation of populations and extinction of species (Harper et al., 2008; Whiteley et al., 2014). Re‐establishing or maintaining historical levels of gene flow by reconnecting populations should be a key component of management and a goal of habitat restoration for species with historically connected populations (Dool et al., 2016; Semlitsch et al., 2017). However, modern anthropogenic landscape changes have made it impossible for some species to naturally recolonize extirpated areas of their historic range and translocations may be necessary to reintroduce species with low vagility.

Together with population structure, estimates of contemporary and historic migration rates are important for estimating connectivity among breeding sites. Historically connected sites with little genetic structure are straightforward candidates for translocations and reintroductions. For species with population structure, moving animals to places where the species has been extirpated or where populations have been severely reduced becomes a more complex challenge. The International Union for Conservation of Nature (IUCN) defines translocations as the human‐mediated movement of living organisms from one area, with release in another. It defines reintroductions as the intentional movement and release of an organism inside its indigenous range from which it has disappeared (IUCN, 2013). Several studies have tested the effectiveness of translocations and reintroductions in order to conserve endangered amphibian species. Eastern hellbenders (*Cryptobranchus alleganiensis*) have survived several years post‐release in sites where they were translocated to supplement existing populations (Kraus et al., 2017; McCallen et al., 2018) and spotted salamanders (*Ambystoma maculatum*) have been reintroduced by moving egg masses into artificial ponds to accelerate restoration without compromising larval survival (Sacerdote, 2009). Overall, promoting dispersal has been a tool in amphibian conservation and may be used to bolster and expand populations of imperiled species.

The reticulated flatwoods salamander (RFS, *Ambystoma bishopi*) is a federally endangered species with a highly fragmented range. Historically, RFS occurred throughout the southeastern United States in fire‐maintained longleaf pine (*Pinus palustris*) ecosystems (Palis, 1996; Petranka, 2010). Over the last several decades, this species has declined for many reasons including climate change, drought, continued loss of flatwoods habitat, and a change in fire regime from summer fires when breeding habitat is dry to prescribed fires in winter when habitat is wet (Bishop & Haas, 2005; Gorman et al., 2013). Winter fires consume less vegetation and leave undesirable plants in breeding habitats, which reduces recruitment and ultimately causes localized extinctions and increased fragmentation. The RFS now only occurs in a fraction of its former range with just six known breeding sites (Farmer et al., 2016; O’Donnell et al., 2017; Semlitsch et al., 2017).

The RFS breeds in ephemeral, fishless ponds dominated by grasses and forbs (Palis, 1996, 1997). These ponds dry during the summer months but fill with rain in the winter (Chandler et al., 2016). During rain events in October and November, adults migrate to dry pond basins and lay their eggs in anticipation of the seasonal inundation (Brooks et al., 2019b; Palis, 1996, 1997). Once the basins fill and the eggs are inundated, the larvae hatch, and eventually metamorphose when the ponds begin to dry in the spring (Palis, 1996). This breeding strategy is highly dependent on predictable seasonal rainfall, and if the ponds do not fill or do not stay full all winter, the entire larval cohort can be lost (Chandler et al., 2016; Palis, 1996).

Breeding sites consist of several separate ponds that are occupied each year and are connected by suitable habitat. Individual salamanders appear to disperse among ponds within a breeding site but do not currently disperse between breeding sites, as estimated from occupancy‐based metapopulation models (Brooks, Smith, Frimpong, et al., 2019) and the lack of suitable connective habitat between breeding sites. Estimates of dispersal in relation to geographical distance have led the United States Fish and Wildlife Service (USFWS) to define any occupied pond (and a 460‐m radius around it) as a breeding population, and any grouping of ponds within 3.2 km (2 miles) of each other as a breeding site (USFWS, 2015). Populations of RFS are typically managed by USFWS at the breeding site level.

Genetic population structure, effective number of breeders, and gene flow have been previously estimated for RFS with microsatellite data, suggesting that there is some structure among ponds within breeding sites (*F*_ST_, 0.004–0.112), limited migration, and a low mean number of breeders per pond (*N*_b_: 12.5–30.1, Wendt et al., 2021). Low N_E_ has also been observed in another endangered amphibian, the California tiger salamander (*Ambystoma californiense*), where N_E_ estimates ranged from 11 to 64 per pond with an average of 30 (Wang et al., 2011). To effectively estimate population structure at small scales, a larger number of genetic markers may be needed since subtle patterns can be obscured when few makers are used (Rittmeyer & Austin, 2015). For example, several hundred single nucleotide polymorphisms (SNPs) were better able to detect fine‐scale structure in tiger salamanders (*Ambystoma tigrinum*) than 12 microsatellites (McCartney‐Melstad et al., 2018; Titus et al., 2014). Furthermore, mitochondrial (mtDNA) data can be included in analyses of population structure and gene flow to examine sex‐biased dispersal. Mitochondrial DNA is inherited via the matrilineal line, and by comparing estimates obtained for mtDNA and nuclear data, it may be possible to elucidate patterns of migration for males and females.

Understanding patterns of gene flow and population structure at the landscape and local scale is necessary to determine the suitability of translocations for the RFS. The aims of this research were to estimate genetic structure, effective population size, genetic variation, and historic and contemporary gene flow among five extant breeding sites of RFS. We generated potential immune gene SNP data, which were complemented by sequence data generated for two major histocompatibility (MHC) exons and one mitochondrial marker in Williams et al. (2020). Our goal was to leverage these three marker types, at the local and landscape scale, to make estimates between and within breeding sites, and to ultimately explore a more complete image of RFS population structure, genetic diversity, gene flow, and sex‐biased dispersal. Immune genes were targeted because pathogens are a major threat to amphibians globally (McCallum, 2007), and variation at these genes is associated with broad resistance to pathogens (Sommer, 2005). These genes may show whether RFS populations have responded to past infections in a way that would create population structure or indicate (through low variation) populations that may be especially vulnerable to disease. Assessing connectivity and genetic differentiation among the remaining breeding sites can inform potential reintroductions and translocations of RFS in order to begin re‐establishing this species across its former range and thus reduce its risk of extinction (Semlitsch et al., 2017).

## 2 METHODS

### 2.1 Sample collection

**FIGURE 1:**
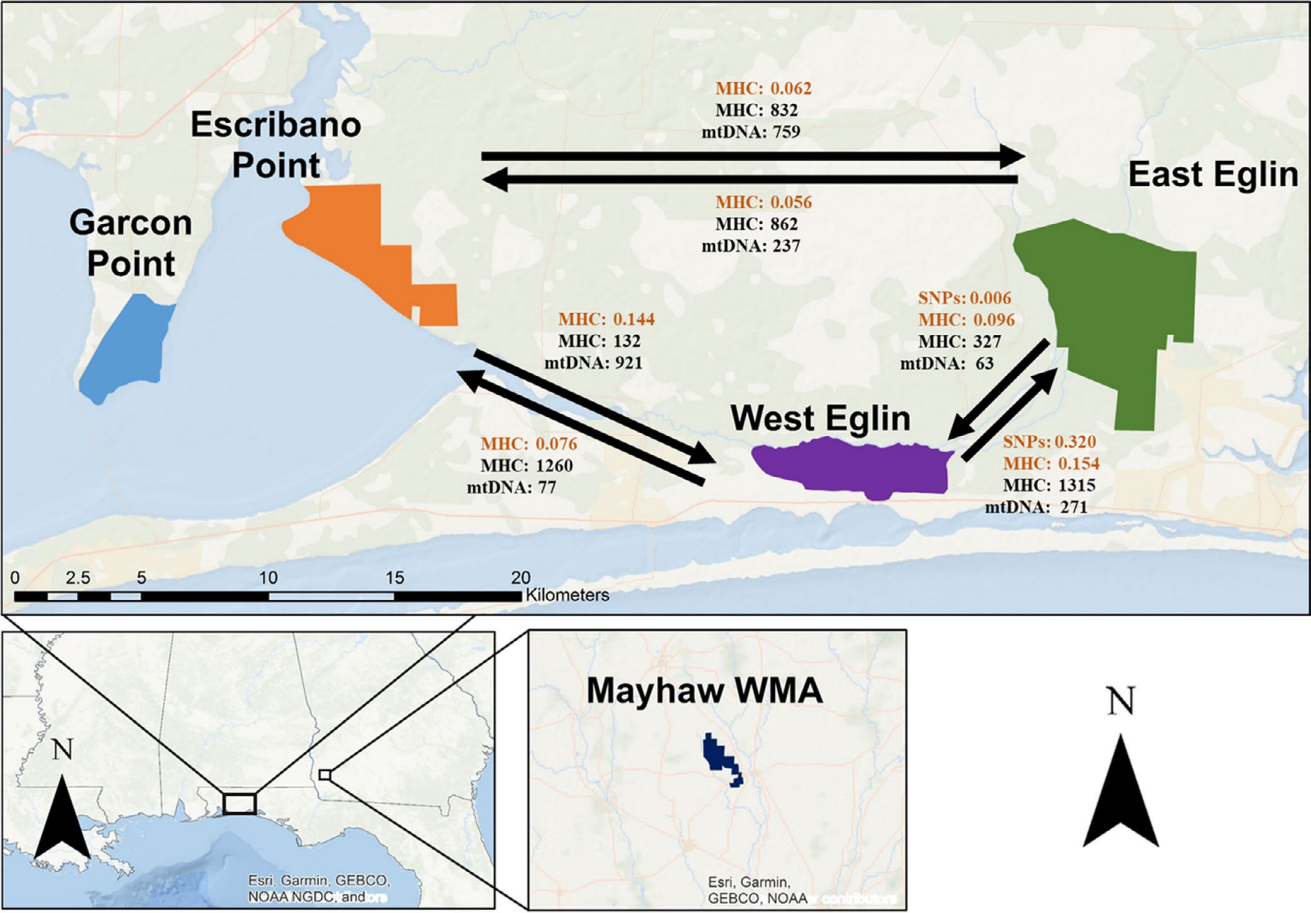
Map of sampled RFS breeding sites, arrows indicate migration rates. Black font: Estimates of mutation scaled migration rate per generation using Migrate‐N. Orange font: BayesAss estimates of migrants per generation. RFS, reticulated flatwoods salamander

Tissue samples were collected from five RFS breeding sites found on public lands (Figure 1) throughout the southeast. Briefly, RFS were collected with dip nets and funnel traps at six ponds on Eglin Air Force base (AFB), Florida, between 2011 and 2017 (Table 1). These six ponds included two separate breeding sites, one in the east (ponds 4, 5, 53, and 212) and one in the west (ponds 15 and 32). In 2018 and 2019, additional RFS samples were collected at five ponds on Escribano Point Wildlife Management Area (WMA), Florida; at one pond at Garcon Point Water Management Area, Florida; and at one pond on Mayhaw WMA, Georgia. Sample sizes at some breeding sites were limited by RFS population size and permission to access sites. For example, only five samples were available from Mayhaw, GA, because this population was only discovered in 2015 and despite surveying, only a few individuals have ever been found at that location. Furthermore, the population at Garcon Point was small at the time of sampling in 2018 and is now thought to be extirpated (pers. comm., Laura Jones and Pierson Hill). More details on the sampling protocol can be found in Williams et al. (2020).

**TABLE 1:** Sample sizes for each genetic marker by breeding site

Site	MHC	mtDNA	SNP
Eglin East	29	143	73
Pond 004	6	51	24
Pond 005	8	54	24
Pond 053	10	24	19
Pond 212	5	14	6
Eglin West	18	30	23
Pond 015	12	24	19
Pond 032	6	6	4
Escribano	28	46	0
Borrow	5	10	0
Cluster	3	11	0
Honey	5	7	0
Ghost	6	9	0
Torpedo	7	8	0
Garcon	4	3	0
Mayhaw	5	5	0
Total	84	227	96

Note

Numbers given for MHC data are the numbers of individuals sequenced at both MHC class Iα and IIβ.

### 2.2 SNP sequencing

DNA was extracted from tissues using a DNEasy Blood and Tissue Kit (Qiagen). Hundreds of RFS immune genes were sequenced in a target enrichment experiment using 19,339 RNA‐biotinylated baits designed to target RFS immune genes. To create custom RNA baits, the axolotl (*Ambystoma mexicanum*) transcriptome (Bryant et al., 2017) was filtered by the gene ontology term “immune response” using the GO2TR 1.0.8 pipeline (Gene Ontology to Target Region, Elbers & Taylor, 2015). This pipeline retained all exons related to immune response (Ortutay & Vihinen, 2006). Using these exons, Arbor Biosciences designed custom 120‐bp baits with 2x tiling to capture RFS immune genes (hereafter, baits).

In 2017, 96 samples from Eglin AFB (east = 73, west = 23) were selected for the target enrichment experiment. Due to sampling constraints, population sizes, and permit access, samples from other sites were not available for the target enrichment experiment. Libraries were prepared using 10 ng of DNA and a KAPA HyperPlus Illumina prep kit in combination with dual‐indexed 8‐bp Illumina adapters (KAPA Biosystems). Libraries were combined to create 16 equimolar pools containing six libraries each. These pooled libraries then underwent sequence capture following the MyBaits version 3.0 protocol (Arbor Biosciences). First, each pool was combined with baits and incubated in solution for 21 h at 65°C. Next pools were washed to retain only “captured” baits and amplified with 14 PCR cycles. Pools were then cleaned with 1× concentration of KAPA pure beads (Indianapolis, IN, USA) to remove excess PCR product and unincorporated baits. Before sequencing, samples were combined into final pools of equimolar concentrations. To achieve adequate coverage, this final pool of 96 enriched libraries was sequenced twice on mid‐output flow cells of an Illumina NextSeq 500 system at Pennington Biomedical Research Center using 75‐bp paired‐end reads.

Raw sequence reads were demultiplexed using the Illumina BaseSpace platform permitting zero mismatches in the barcodes. Adapter quality trimming was performed on demultiplexed paired‐end reads using all adapters sequences supplied with BBDuk 38.16 (https://sourceforge.net/projects/bbmap/) and the following options: ktrim = right, which trims kmers from 5′ to 3′ direction of a read, *k* = 23 which sets the kmer length to 23 bp, mink = 11 which sets the minimum kmer matching length to 11, hdist = 1 that uses a hammering distance of one to increase the number of kmers stored, tpe which trims both reads to the same length, tbo which trims adapters based on pair overlap, qtrim = rl for quality trimming right and left, and trimq = 15 which sets the quality trim to 15 using the Phred algorithm. Next, quality and adapter trimmed paired‐end reads were mapped to the axolotl 3.0.0 reference genome (https://axolotl‐omics.org/dl/AmexG_v3.0.0.fa.gz) using BBMap 38.16 (https://sourceforge.net/projects/bbmap/) and the vslow and usejni options. SAM files were converted to BAM files with SAMtools 1.9 (Li et al., 2009). Picard 2.18.10 (http://broadinstitute.github.io/picard) was used to clean, sort, add read groups, and mark duplicates. The program CallVariants 38.16 (https://sourceforge.net/projects/bbmap/) was used to call SNPs by ignoring duplicates and keeping SNPs with quality scores greater than or equal to 27. Finally, this dataset was reduced to only di‐allelic SNPs with genotype missingness of 5% or less were retained using VCFtools 0.1.15 (Danecek et al., 2011).

Retained SNPs were tested for linkage disequilibrium and Hardy–Weinberg equilibrium (HWE) using VCFtools, geno‐chisq, and hardy tests. The p.adjust function in R‐4.0.2 (R Core Team, 2018) was then used to correct for multiple tests using the false discovery rate (FDR, Benjamini & Hochberg, 1995). Gene duplication in the RFS, a pattern observed in other amphibians (McCartney‐Melstad et al., 2018; Waples, 2015), can lead to heterozygote excess causing *H*_O_ >> *H*_E_. This possible gene duplication prompted filtering of the SNP data using stringent conditions. Any SNP that had a FDR less than 0.05 in one or more ponds was discarded from the dataset. Next, BayeScan 2.1 (Foll & Gaggiotti, 2008) was used to predict if SNPs were putatively under selection. As no SNPs were identified as being under selection, SNPs were used to estimate population structure and gene flow under neutral expectations. Finally, blastn v. 2.2.31+ (Altschul et al., 1990) was used to identify bait locations in the axolotl genome, and custom gene annotations were used to predict the genes each SNP might occur in (Sergej Nowoshilow, pers. comm.).

Allelic richness (AR), observed (*H*_O_), expected heterozygosity (*H*_E_), and pairwise *F*_ST_ (Weir & Cockerham, 1984) were estimated at the pond and breeding site level for SNP data using hierfstat v. 0.04–22 (Goudet, 2005). Heirfstat was also used to conduct principal component analysis (PCA) for SNP data. GenePop v4.6 (Raymond & Rousset, 1995; Rousset, 2008) was used to calculate the inbreeding coefficient for each breeding site (F*_IS_*, Weir & Cockerham, 1984) along with an isolation by distance analysis (IBD). IBD was conducted using the decimal degree location for the center of each pond, 10,000 Mantel test permutations, and a minimum distance of 0.001 decimal degrees between samples. Effective population size was calculated for each breeding site using the linkage disequilibrium method in program NeEstimator v2.1 (Do et al., 2014.). Recent patterns of gene flow were estimated with BayesAss 3.0 (Wilson & Rannala, 2003). BayesAss estimates migration rate over the last three generations by calculating the probability of migrant ancestry for all individuals, assigning them as either a non‐migrant, 1^st^ generation, or migrants that are 2^nd^ generation or greater. For all analysis, 3 × 10^6^ iterations were run starting with a burn‐in of 2 × 10^6^ followed by 1 × 10^6^ iterations with a sample taken every 100 steps for a posterior dataset of 1 × 10^4^ samples. These analyses were repeated 10 times using different seeds to ensure convergence of models. STRUCTURE v2.3.4 (Pritchard et al., 2000) and Admixture 1.3.1 (Alexander et al., 2009) were run to assess population admixture using ancestry models to infer the number of populations (*K*) from the data. In program Admixture, the lowest cross‐validation value was used to select *K* between *K* = 1 to *K* = 7. For STRUCTURE, the model that maximizes marginal likelihood was used and results were confirmed by comparing distruct plots (Rosenberg, 2003).

### 2.3 MHC and mtDNA sequencing

To include other breeding sites and multiple marker types for population structure analyses, we used sequence data generated in Williams et al. (2020) for major histocompatibility complex (MHC) class Iα exon 3 and class IIβ exon 2 and the mitochondrial control region (Williams et al., 2020). An analysis of molecular variance (AMOVA) was conducted for all sampling locations with MHC and mtDNA sequence data using Arlequin 3.5.2.2 (Excoffier et al., 2005). Samples from Mayhaw and Garcon were removed from migration, STRUCTURE, and IBD analyses because of small sample sizes.

For MHC data, pairwise *F*_ST_ (Weir & Cockerham, 1984) was calculated in hierfstat v.0.04–22, where MHC sequences were coded as alleles (i.e., each distinct sequence at a locus was given a number, e.g., allele 01). MHC data were only used at the site level because sample sizes at individual ponds were low (≤12) and the SNP data provided a more robust analysis for nuclear data at the smaller spatial scale. For mtDNA, Φ_ST_ was calculated in Arlequin 3.5 using mitochondrial haplotype frequencies. Unlike the MHC data, this analysis was conducted at both the pond and breeding site level.

Principal component analysis, effective population size, and program STRUCTURE v2.3.4 (Pritchard et al., 2000) were run for MHC markers as described for SNP data, but these analyses were not implemented for mitochondrial DNA because they require diploid data. GenePop v4.6 was used to estimate the inbreeding coefficient and isolation by distance. Recent migration rates between breeding sites (Eglin East, Eglin West, and Escribano) were estimated using MHC and mitochondria haplotypes in the program BayesAss 3.0 using the conditions described above for SNPs. Historic migration rates (>5 generations) were estimated by Bayesian inference in Migrate v3.7.2 (hereafter Migrate‐N, Beerli, & Felsenstein, 2001). This program estimates two metrics, the mutation scaled population size (ϴ), which is calculated as ϴ = 4*N*_e_ *μ* where *N*_e_ is effective population size and *μ* is mutation rate, and; a mutation scaled migration rate measured as the number of migrants per generation calculated as *m*/*μ* where *m* is the migration rate and *μ* is the mutation rate. Here, a full migration matrix model was used, which assumes direct gene flow between all populations. After testing multiple parameters, three long chains were run with a burn‐in of 5 × 10^6^ iterations and, to avoid autocorrelation, after the burn‐in samples were taken every 50 steps for 5 × 10^4^ iterations for a posterior dataset of 1 × 10^4^ sampling iterations.

## 3 RESULTS

### 3.1 SNP sequencing

After quality control filtering, 263,127,494 reads were assignable to all individuals with 2,740,911 ± 2,328,524 (mean ± SD) reads assigned per individual (range = 477,444–14,876,290). Nine percent of the original reads were removed as PCR or optical duplicates, and 95% of the remaining reads were successfully aligned to the axolotl genome (2.27 × 10^8^ reads). After alignment and comparison to the axolotl genome, 183 SNPs were identified, but after filtering, only 90 were polymorphic, in HWE, and in linkage equilibrium. All further analyses were conducted using these 90 SNPs.

Many sequencing reads were off‐target: on average, only 1.39% (min–max; 0.32–2.26%) of reads were on‐target. For this analysis, on‐target and off‐target reads were included when coverage permitted. Numerous RFS reads mapped to regions of the axolotl genome that are not predicted to represent immune genes while others could not be mapped at all. This issue may be caused by the phylogenetic distance between RFS and axolotls. Although they are members of the same genus, these two species likely diverged about 20 million years ago (Hime et al., 2021; Williams et al., 2013). Consequently, we consider the SNPs generated to be anonymous.

### 3.2 Genetic variation and effective population size

In the SNP dataset, allelic richness was 1.38 at East Eglin and 1.44 at West Eglin (Table 2). At East Eglin and West Eglin, *H*_O_ was 15% and 18%, respectively, while *H*_E_ was 12% and 15%, respectively (Table 2). Previous work shows that genetic variation was low at both MHC exons (Table 2, Williams et al., 2020). For class Iα, only three alleles were found with a nucleotide diversity of 0.001, *H*_E_ of 19.9%, and a *H*_O_ of 16.8% (Williams et al., 2020). MHC class IIβ had five alleles with a nucleotide diversity of 0.004, *H*_E_ of 53.7%, and *H*_O_ of 34.4% (Williams et al., 2020). Higher levels of *H*_E_ and a *H*_O_ at MHC versus SNP loci is likely a function of the marker type, as SNP markers represent a single base pair in the genome (with two possible alleles) whereas MHC exons represent several hundred base pairs that have the possibility of generating many types of alleles and heterozygotes.

**TABLE 2:** Allelic richness (AR), observed heterozygosity (*H*_O_), expected heterozygosity (*H*_E_), and inbreeding coefficient (*F*_IS_) at Eglin Air Force Base using SNP and MHC data (MHC data originally presented in Williams et al., 2020)

Marker	Site	*n*	AR	*H*_O_	*H*_E_	*F*_IS_
SNP	Pond 004 (East)	24	1.36	0.13	0.11	−0.22
	Pond 005 (East)	24	1.41	0.16	0.14	−0.22
	Pond 053 (East)	19	1.37	0.14	0.12	−0.24
	Pond 212 (East)	6	1.4	0.17	0.14	−0.29
	Pond 015 (West)	19	1.43	0.17	0.14	−0.23
	Pond 032 (West)	4	1.66	0.19	0.16	−0.22
	East Eglin	73	1.38	0.15	0.12	−0.18
	West Eglin	23	1.44	0.18	0.15	−0.17
MHC Iα	East Eglin	105	1.39	0.11	0.12	0.11
	West Eglin	37	1.65	0.24	0.22	−0.12
	Escribano	39	2.84	0.59	0.64	0.08
	Mayhaw	5	2.00	0.60	0.56	−0.09
	Garcon Point	4	1.00	0.00	0.00	—
MHC IIβ	East Eglin	29	2.25	0.38	0.54	0.30
	West Eglin	26	2.51	0.35	0.52	0.34
	Escribano	29	2.28	0.28	0.53	0.48
	Mayhaw	5	2.00	0.60	0.47	−0.33
	Garcon Point	4	2.00	0.25	0.25	0.00

At East Eglin, West Eglin, and Escribano, *F*_IS_ for MHC class IIβ was 0.30, 0.34, and 0.48 respectively (Table 2). Contrastingly, *F*_IS_ for MHC class Iα was lower at these three sites with values of 0.106, −0.120, and 0.084. Nine mitochondrial haplotypes were observed, two of which had previously been described by Pauley et al. (2007; GenBank accession numbers H2: EU517607.1 and H3: EU517606.1) while the other seven were previously undescribed. Of the nine haplotypes, one occurred at all breeding sites (H3) while two others (H2, H9) were found at multiple breeding sites (Figure 1, Williams et al., 2020).

Effective population size could not be reliably estimated with MHC data, as values were either infinite or the 95% confidence intervals (CI) overlapped with 0 (Table S1). Infinite values indicate no genetic variation caused by drift and can be explained by sampling error or limited genetic variation (Waples & Do, 2010). Effective population size could be calculated at the site level with SNP data using a lowest allele frequency of <0.010. For East Eglin, N_E_ was 206.3 (CI: 164.2–273.5) while N_E_ for West Eglin was smaller at 120.2 (CI: 74.2–290.2, Table S1).

### 3.3 Population structure

Pairwise *F*_ST_ was estimated with SNP and MHC data for Eglin and pairwise *F*_ST_ was estimated with MHC data for Escribano. Using the SNP dataset for individual ponds within breeding sites on Eglin, all pairwise *F*_ST_ were ≤0.056 while *F*_ST_ between Eastern and Western Eglin was 0.004 (Table 3). No pattern of isolation by distance was detected when comparing all ponds on Eglin (*p* = 0.113). For MHC loci, *F*_ST_ values between breeding sites ranged from −0.007 to 0.091 (Table 4). MHC *F*_ST_ values between ponds within breeding sites could not be calculated due to small sample sizes at individual ponds.

**TABLE 3:** *F*_ST_ calculated with SNP data and Φ_ST_ calculated with mtDNA data by pond on Eglin

	Pond 004 [East]	Pond 005 [East]	Pond 053 [East]	Pond 212 [East]	Pond 015 [West]	Pond 032 [West]
Pond 004 [East]	—	0.46	0.28	0.48	15.67	15.54
Pond 005 [East]	0.007 (0.033)	—	0.36	0.94	15.45	15.32
Pond 053 [East]	0.002 (−0.018)	0.012 (**0.065**)	—	0.68	15.4	15.26
Pond 212 [East]	0.005 (0.068)	0.007 (**0.202**)	0.013 (0.025)	—	15.9	15.77
Pond 015 [West]	0.006 (**0.396**)	0.0002 (**0.401**)	0.006 (**0.427**)	0.001 (**0.559**)	—	0.14
Pond 032 [West]	**0.042** (**0.549**)	0.021 (**0.541**)	**0.056** (**0.608**)	**0.031** (**0.874**)	0.018 (0.057)	—

Note

*F*_ST_ and Φ_ST_ values are below the diagonal and approximate Euclidean distance (km) is given above the diagonal. *F*_ST_ values calculated with SNP data are listed first and the mtDNA Φ_ST_ values are given in parentheses. Bolded values are significant at *p* < 0.05. Pairwise *F*_ST_ between the two breeding sites was 0.004.

**TABLE 4:** *F*_ST_ calculated with MHC data and Φ_ST_ calculated with mtDNA data by site

	Eglin East	Eglin West	Escribano
Eglin East	—	13.01	30.17
Eglin West	−0.007 (**0.356**)	—	19.64
Escribano	**0.091** (**0.152**)	**0.082** (**0.354**)	—

Note

*F*_ST_ and Φ_ST_ values are below the diagonal and approximate Euclidean distance (km) is given above the diagonal. *F*_ST_ values calculated with MHC data are listed first and the mtDNA Φ_ST_ values are given in parentheses. Bolded values are significant at *p* < 0.05.

The mitochondrial control region was used to estimate Φ_ST_ between individual ponds within Eglin or Escribano and between the three breeding sites. For ponds within East Eglin, Φ_ST_ values ranged from −0.169 to 0.203 while the pairwise comparison between the two West Eglin ponds was 0.057 (Table 3). On Escribano, Φ_ST_ values were between −0.063 and 0.420 (Table 5). Mitochondrial data did show a pattern of isolation by distance at some sites. When comparing ponds within East or West Eglin separately, there was no isolation by distance (IBD *p* = 0.173 and *p* = 0.663, respectively) but when comparing East and West Eglin there was isolation by distance (IBD *p* = 0.014). Furthermore, there was a pattern of isolation by distance (IBD *p* = 0.036) for ponds on Escribano. When individual ponds within breeding sites were examined more closely, the highest Φ_ST_ values (0.280–0.420) were between ponds separated by more than 1.5 km. Ponds less than 0.9 km apart had Φ values below 0.07. Finally, between the breeding sites of East Eglin, West Eglin, and Escribano, Φ_ST_ ranged from 0.152 to 0.356 (Table 4).

**TABLE 5:** Φ_ST_ calculated with mtDNA data by pond on Escribano

	Borrow	Cluster	Honey	Ghost	Torpedo
Borrow	—	1.01	3.40	3.30	2.80
Cluster	−0.063	—	2.34	2.28	2.33
Honey	**0.354**	**0.400**	—	0.130	0.530
Ghost	0.287	**0.361**	−0.087	—	0.630
Torpedo	**0.386**	**0.420**	−0.147	−0.030	—

Note

Φ_ST_ values below the diagonal and approximate Euclidean distance (km) above the diagonal. Bolded values are significant at *p* < 0.05.

**FIGURE 2:**
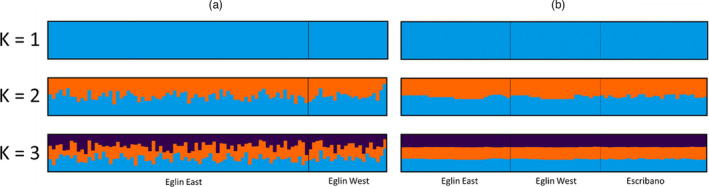
Distruct plots for SNP (a) and MHC class Iα and IIβ (b) data. For *K* = 2 and 3, individual membership does not align with sampling location indicating a lack of population structure and supporting *K* = 1. Distruct plots of SNP data broken down by pond found in Figure S3. MHC, major histocompatibility complex; SNP, single nucleotide polymorphisms

AMOVA results with MHC data indicated little population structure: Only 9.53% of the total variation was among breeding sites (Table 6). Greater population structure was detected with mitochondrial data: 25.49% of the total variation was among breeding sites (Table 6). Program STRUCTURE cannot calculate Δ*K* when *K* = 1, so to investigate *K* = 1–7, we used distruct plots as well as log likelihoods to determine *K*. With the SNP dataset, STRUCTURE suggested a single population (*K* = 1) on Eglin. This result was confirmed by comparing distruct plots of *K* = 1–3 (Figure 2). Program Admixture also suggested a single population on Eglin based on the cross‐validation (CV) values for *K* = 1–7 in the SNP and MHC datasets (Table S2). For MHC data at Eglin and Escribano, program STRUCTURE suggested two populations using ΔK, but one population using log‐likelihood values. To further evaluate *K* = 2, distruct plots were compared (Figure 2). *K* = 2 showed no distinct patterns between populations and so *K* = 1 was accepted. These results are supported by the PCAs obtained for both SNP and MHC data (Figures S1 and S2). For the PCAs, SNP data clustered in a single group regardless of collection location or dataset, and MHC data showed no discernable pattern based on sampling site.

**TABLE 6:** AMOVA as estimated with MHC and mtDNA data

Markers	Source of variation	Degrees of freedom	Sum of squares	Percentage Variation
MHC Iα & IIβ	Among breeding sites	4	8.42	9.53
	Among individuals within breeding sites	85	46.86	19.53
	Within individuals	90	32	70.95
mtDNA	Among breeding sites	4	14.56	25.49
	Within individuals	233	67.97	74.51

Note

All estimated values are significant.

### 3.4 Gene flow

For program Migrate‐N, the mode migration estimate for SNP, MHC, and mitochondrial data was reported, because for SNP and MHC markers, the mean, median, and mode were almost identical (Table S3), but for mitochondrial estimates, the mode better represented the peak of the distribution on the curve (Figures S4–S6). For mitochondrial data, migration rates were not multiplied by four (to account for mtDNA N_E_) to equate them with migration rates for a nuclear marker; instead, they were left unadjusted to estimate migration for this haploid, maternally inherited marker. Migrate‐N estimates had wide posterior distributions for both migration and ϴ estimates. For SNP data, migration rates for East Eglin to West Eglin were 10.1 and 1.2 from West to East (Table S3 and Figure S4). Migration rates for MHC class IIβ were highest from East to West Eglin at 1315.8 with West to East migration rates of 372.5. Migration rates for West Eglin to Escribano were 1260.8, while Escribano to West Eglin were 132.5. Migration rates between East Eglin and Escribano were moderate and almost equal in both directions at 832.5 and 862.5 (Table S3 and Figure S5). For mitochondrial data, models gave wide estimates with long tails. Estimates of migration with mitochondrial data were highest from Escribano to East and West Eglin at 759.0 and 921.0, respectively, while migration out of West Eglin was the lowest at 63.0 to East Eglin and 77.0 to Escribano (Table S3 and Figure S6). Although the lowest confidence intervals for many mitochondrial migration rates were at or near zero, mean, median, and mode estimates were always more than 60 individuals. MHC class Iα estimates were uninformative, probably because range‐wide genetic variation was very low.

Migration rates as estimated with SNP data in BayesAss showed an asymmetrical pattern: 32.0% of individuals in the West were migrants from the East while 0.6% of individuals in the East were migrants from the West (Table S4). BayesAss estimates for MHC data were generally lower than estimates obtained with SNP data. West Eglin had the most individuals of migrant ancestry (14.4–15.4%) while East Eglin and Escribano had fewer individuals of migrant ancestry (5.6–8.8%; Table S4).

## 4 DISCUSSION

Reticulated flatwoods salamanders showed little population structure and some gene flow across the core of their remaining range as estimated with SNP data; however, due to the discordant results obtained for nuclear and mitochondrial markers, our results suggested males drive dispersal as analyses with mtDNA data demonstrated structure and isolation by distance. At a fine geographical scale among ponds on Eglin and among ponds on Escribano, nuclear markers showed gene flow and little population structure, a result that is consistent for all analyses (*F*_ST_, PCA, STRUCTURE, IBD, and Admixture). This finding is supported by the AMOVA results, which showed that the majority of genetic variation occurs within individuals and only 9.5% of variation was explained among breeding sites. This result is lower, but similar, to previous research on Eglin, which estimated structure with microsatellite data (within East Eglin mean *F*_ST_ = 0.055 (range = 0.009–0.112); within West Eglin mean *F*_ST_ = 0.035 (range = 0.004–0.079), between East and West Eglin = 0.104 (range = 0.061–0.163, Wendt et al., 2021). Although estimates obtained with microsatellite, SNP, and MHC markers cannot be compared directly, an examination of overall patterns is useful. *F*_ST_ estimates between ponds within East Eglin or within West Eglin are low for both microsatellite and SNP data. However, microsatellite *F*_ST_ values are higher between the two Eglin breeding sites than the *F*_ST_ values generated with SNP or MHC data. In contrast to nuclear markers, mtDNA showed structure among some ponds on Escribano and between East and West Eglin (Tables 4 and 5).

At a broad geographical scale, the combined data from ponds within each breeding site (i.e., East Eglin, West Eglin, and Escribano) continued to support gene flow and a lack of genetic structure as estimated with SNP and MHC data. However, structure was high between all breeding sites as estimated with mitochondrial DNA (Table 4). The disparity in genetic structure and dispersal estimates for nuclear and mitochondrial DNA is indicative of male‐biased dispersal, a pattern observed in other amphibians including the red‐backed salamander (*Plethodon cinereus*) and the alpine salamander (*Salamandra atra*, Helfer et al., 2012, Liebgold et al., 2011). Females may be capable of moving long distances, but as fall breeders, they rely on cues like previous experience to select suitable egg laying habitat. As a result, they may have a stronger philopatric connection to their natal pond in contrast to males that orient based on receptive females. Thus, females do not appear to disperse as widely given the pressure to select suitable egg laying habitat (Burkhart et al., 2017; Moore & Whiteman, 2016; Peterman et al., 2015).

Although there was some mitochondrial genetic structure over short distances, analyses indicated that Φ_ST_ increased considerably when ponds were separated by more than 1.5 km (Tables 3 and 4), a value similar to the dispersal distances calculated using spatially explicit occupancy models (Brooks, Smith, Frimpong, et al., 2019). This was apparent on Eglin where IBD analysis of mitochondrial data showed no relationship between distance and structure within breeding sites, but did show a significant correlation between East and West Eglin (Tables 3 and 4). In contrast, MHC and SNP data showed no isolation by distance within or between Eglin sites indicating that males may move, or historically moved, between these ponds at a higher rate than females. Notably, although Escribano is treated as one breeding site by the USFWS, mitochondrial and IBD data showed that highly philopatric females may effectively occupy two distinct breeding sites at this location (Table 5): Borrow and Cluster ponds, which are separated by approximately 2.3 kilometers from the second group of ponds, Honey, Torpedo, and Ghost. However, it is difficult to estimate the precise relationship between dispersal and distance because distances between sampled ponds have a clumped distribution (currently occupied ponds are either 0.1–1.5 km apart, 2.5–3.5 km apart, or >10.0 km apart) and do not fall on a continuum. Mitochondrial data suggested that females typically do not disperse more than 1.5 km but that may be an underestimate because there were no sampled ponds in the 1.5–2.5 km range.

Other studies have found greater population structure in fall breeding ambystomatids, such as the RFS, than in spring breeders on the same landscape. For example, ringed salamanders (*Ambystoma annulatum*) and marbled salamanders (*Ambystoma opacum*), both fall breeders, showed more population structure on Fort Leonard Wood, MO than spotted salamanders, which breed in the spring (Burkhart et al., 2017, Peterman et al., 2015). Fall breeders may show increased philopatry to breeding ponds because they mate when ponds are dry and must deposit eggs in the dry basin. Accordingly, selecting suitable egg laying habitat is more difficult because females must anticipate inundation as compared to the spring where water is present when breeding begins. Consequently, for females, there is a strong selective pressure to choose breeding sites based on cues such as previous experience or pond‐associated vegetation (Brooks, Smith, Frimpong, et al., 2019). This pressure to select breeding sites may contribute to greater philopatry in fall breeding salamanders thus decreasing gene flow and increasing genetic structure (Burkhart et al., 2016, Peterman et al., 2015).

We attempted to isolate SNPs from immune genes, and although most were off‐target and effectively anonymous, it is possible that selection may play a role in population structure despite our BayeScan results indicating SNP neutrality. However, had there been selection on specific SNPs with different disease challenges at the various sampling sites, we would expect to find population structure rather than its absence. It would seem unlikely that 90 SNPs would all be under selection in the same way at multiple locations so as to cause a consistent lack of population structure across sampling sites. Future work with a larger number of SNP loci located throughout the genome may help to clarify population structure in RFS.

Measures of SNP genetic diversity were low and mostly uniform across East and West Eglin. MHC data showed that Escribano had the most diversity whereas East Eglin and Garcon Point had less than the other sites (Williams et al., 2020). Allelic richness, observed heterozygosity, and expected heterozygosity were all lower than a similar endangered amphibian, the California tiger salamander. Using 11 microsatellites across multiple California tiger salamander breeding ponds, all measures of genetic diversity were higher than the RFS (AR =15.6–24.0, *H*_O_ = 0.70–0.92, *H*_E_ = 0.75–0.89, Wang et al., 2011). Despite lower estimates of diversity, estimates of effective population size were similar between the RFS and California tiger salamander. Using SNP data, N_E_ for the RFS was 206.3 (164.2–273.5) at East Eglin and 120.2 (74.2–290.2) at West Eglin. For the California tiger salamander, N_E_ was 203 when summed across 10 breeding ponds (Wang et al., 2011). Although estimates obtained with microsatellites cannot be directly compared with SNP and MHC markers, it appears that diversity is low across the RFS’s sampled range, but N_E_ may be comparable to other endangered ambystomatids.

F_IS_ values were below zero for SNP data (Table 2) in contrast to MHC *F*_IS_ values, which ranged up to 0.48 (Williams et al., 2020). The larger SNP dataset may have provided a more precise estimate of inbreeding, but additionally, selection for specific MHC alleles caused by disease history may have reduced genetic diversity and increased F_IS_ in that region without affecting the SNP regions (Williams et al., 2020).

Data from all marker types suggest that some migration occurs, or historically occurred, between each of the East Eglin, West Eglin, and Escribano breeding sites. Notably, MHC markers had much higher estimated migration rates compared to SNP data (MHC = 132–1315, SNP = 1.2–10.1). The disparity between these estimates may be caused by similar selective pressures at MHC genes, which could inflate migration estimates. Given the considerable distance between East and West Eglin (13 km), and limited mobility for salamanders, it is likely that MHC data overestimated the amount of migration between these distant sites. Nevertheless, migration estimates generated from our datasets suggest that some migration, though perhaps limited, has occurred between East Eglin, West Eglin, and Escribano.

Both Migrate‐N (mtDNA) and BayesAss (SNP and MHC) suggest that West Eglin receives more immigrants from and contributes fewer emigrants to Escribano and East Eglin overall. Asymmetrical gene flow is most pronounced on Eglin with an approximate 3:1 migration rate from East to West Eglin (Migrate‐N) as estimated with all three marker types. This uneven gene flow could have biological implications, chiefly that West Eglin may have been a population sink in the past. Because West Eglin also has a smaller N_E_ than East Eglin, it may have lower recruitment than East Eglin, leading to more immigration and less emigration. However, this asymmetrical migration rate could be an artifact of different demographic histories on each site. For instance, directional selection at immune genes (especially MHC) could skew allele frequencies and inflate migration estimates. Alternatively, a more severe bottleneck at one site may have removed genetic variants found at other sites, creating an uneven pattern of diversity that may be interpreted as asymmetrical migration (Wilson & Rannala, 2003). However, it is possible that asymmetrical gene flow is real and caused by topographical conditions that skew migration patterns, for example, directional water flow that may influence larval dispersal during flooding events, a possibility that requires further study.

Managing all RFS populations in a metapopulation context, in which there is limited gene flow among breeding sites, is an option that may maintain genetic diversity over the long term. In theory, as different alleles drift to fixation in individual breeding sites, global heterozygosity is effectively frozen in the total population, but there is still sufficient gene flow to prevent inbreeding over the long term within breeding sites and spread advantageous alleles across the species’ range (Allendorf et al., 2013). In practice, this approach is most suitable when (1) the population size of individual breeding sites is large enough to avoid inbreeding over the short‐term; (2) there are enough breeding sites for different alleles to randomly fix without being lost in the total population; and (3) there is evidence that the species has evolved in a metapopulation context. In RFS, these conditions arguably do not exist: some populations are very small (e.g., Mayhaw WMA and Garcon Point) and may experience inbreeding over the short term, reducing survival and reproductive success in an endangered species, if gene flow among breeding sites remains low. Moreover, very few breeding sites (*n* ≈ 6) exist, reducing the likelihood that all alleles will be present in at least one breeding site as they drift to fixation, and increasing the risk that stochastic events could reduce the remaining diversity. Finally, the SNP, MHC, and mitochondrial results obtained here showed little population structure and, at least historically, some gene flow among Escribano, East Eglin, and West Eglin breeding sites. This suggests that RFS may not have evolved in a metapopulation scenario with little gene flow among breeding sites. For these reasons, the historic connection among breeding sites might be maintained to facilitate gene flow, thereby reducing genetic drift and inbreeding at individual breeding sites, although we acknowledge that there are other considerations such as limiting the potential spread of disease or undermining currently stable populations. This historic connection could be maintained by expanding suitable habitat or hydrologic management and must take into consideration the population size of donor and recipient populations. However, gene flow among populations may be important given the evident low diversity at immune genes and potential limited ability to combat infections in RFS (Williams et al., 2020). At this time, it is important to maintain and expand current breeding sites to improve habitat, increase population size as well as facilitate gene flow. In future, managing RFS breeding sites as metapopulations may be a better option if population sizes recover sufficiently to limit inbreeding over the short term, and enough breeding sites have been re‐established to ensure that all alleles are likely to be present in the total population as they become randomly fixed in individual breeding sites.

Overall, RFS in this area may have existed as a series of connected populations rather than several discrete metapopulations. Given historic gene flow and a lack of population structure, carefully considered reintroductions could be used as a method to help re‐establish RFS populations on historically occupied sites that are now extirpated (Palis et al., 1997). Several of these extirpated sites are being restored to ensure the survival of RFS in future with the removal of undesirable vegetation, protection of core areas from habitat loss, and a return of important summer fires (pers comm. Kelly Jones and Charlie Abeles). Recolonization of extirpated sites is unlikely to occur without human aid as they are too far from currently active sites to naturally re‐establish, especially because females appear unlikely to disperse across long distances. Even if male salamanders disperse to unoccupied sites, they are unlikely to encounter females. However, RFS reintroductions could move both sexes and begin to expand the limited range of this salamander to ensure its long‐term resilience.

These reintroductions could initially be conducted in locations far from existing RFS populations but using multiple source ponds whose hydrology is similar to the reintroduction location. This approach would minimize the risk of spreading disease and perhaps preserve any local adaptations to environmental variables such as hydroperiod (Richter‐Boix et al., 2015; Wendt et al., 2021). Sites further from the Gulf of Mexico should be prioritized for reintroduction in order to minimize the risks posed by saltwater intrusion associated with hurricanes and sea level rise.

In conclusion, at a broad geographical scale, RFS populations showed little population structure with nuclear SNP data (*F*_ST_ = 0.00–0.09) but higher structure with mitochondrial DNA (Φ_ST_ = 0.15–0.36). At this same scale, mitochondrial DNA exhibited isolation by distance, whereas nuclear markers did not. These discordant results suggest that males drive dispersal whereas females are more philopatric. Using the distance between sampled sites, and sharp increases in Φ_ST_, it is likely that females disperse less than 1.5 km from their natal pond (Tables 3, 4, 5). Finally, program Migrate‐N and BayesAss supported some migration, at least historically, between East Eglin, West Eglin, and Escribano breeding sites.

Future amphibian conservation will require both molecular and traditional conservation work to inform appropriate management strategies, especially in fragmented or shrinking habitats with increasingly less connected populations and fewer active breeding sites. Combining molecular methods with traditional conservation techniques can help identify appropriate source populations for reintroductions, identify sex differences in gene flow, and preserve potentially unique diversity and adaptive potential. Finally, time is a resource that is in short supply: the increasing frequency of strong hurricanes (e.g., Hurricane Michael in 2018 and Hurricane Sally in 2020) as well as the risk posed by global amphibian diseases and inbreeding in small populations underlines the need to begin re‐establishing populations in order to decrease the risk of extinction.

## CONFLICT OF INTEREST

The authors have no conflict of interest for this article.

## Supporting information

Supplementary Material

## Data Availability

MHC and mtDNA data for this study are available at the NCBI GenBank repository (MT475763‐MT475765 and MT425246‐MT425250). Next‐generation sequencing data for this study are available at the NCBI Sequence Read Archive, BioProject, PRJNA750462.
