# Supplementary material for: Population structure, gene flow, and sex‐biased dispersal in the reticulated flatwoods salamander (Ambystoma bishopi): Implications for translocations
Source: Evol Appl. 2021 Aug 27;14(9):2231–43. doi: 10.1111/eva.13287 (PMC8477597; doi:10.1111/eva.13287)
Supplement: Supplementary file 1 — Supplementary Material [file EVA-14-2231-s001.docx]

Table S1. Effective population sizes using the linkage disequilibrium method (NeEstimator v2.1). Mean estimates are on the first line for each population, with 95% confidence intervals underneath in brackets. Infinite values indicate no genetic variation caused by drift and can be explained by sampling error or limited genetic variation (Waples and Do 2010).

| Marker Type | Site | Lowest Allele Frequency | | | |
| --- | --- | --- | --- | --- | --- |
|  |  | 0.050 | 0.020 | 0.010 | 0.000 < 0.010 |
| SNPs | East Eglin | Infinite  (Infinite – Infinite) | Infinite  (2982.6 – Infinite) | 1117.6  (458.9 – Infinite) | 206.3  (164.2 - 273.5) |
|  | West Eglin | Infinite  (Infinite - 271.7) | 120.2  (74.2 - 290.2) | 120.2  (74.2 - 290.2) | 120.2  (74.2 - 290.2) |
| MHC | East Eglin | Infinite  (0.1 – Infinite) | Infinite  (0.1 – Infinite) | Infinite  (0.1 – Infinite) | Infinite  (0.1 – Infinite) |
|  | West Eglin | 4.7  (0.0 – Infinite) | 4.7  (0.0 – Infinite) | 4.7  (0.0 – Infinite) | 4.7  (0.0 – Infinite) |
|  | Garcon | 0.5  (0.0 – Infinite) | 0.5  (0.0 – Infinite) | 0.5  (0.0 – Infinite) | 0.5  (0.0 – Infinite) |
|  | Mayhaw | Infinite  (Infinite – Infinite) | Infinite  (Infinite – Infinite) | Infinite  (Infinite – Infinite) | Infinite  (Infinite – Infinite) |
|  | Escribano | Infinite  (0.9 – Infinite) | Infinite  (0.9 – Infinite) | Infinite  (1.4 – Infinite) | Infinite  (1.4 – Infinite) |

Table S2. Cross validation (CV) values for Admixture for K=1 – 7 using SNP dataset.

| Populations | Cross validation |
| --- | --- |
| K = 1 | 0.27783 |
| K = 2 | 0.31485 |
| K = 3 | 0.35503 |
| K = 4 | 0.38147 |
| K = 5 | 0.40472 |
| K = 6 | 0.41595 |
| K = 7 | 0.42432 |
|  |  |


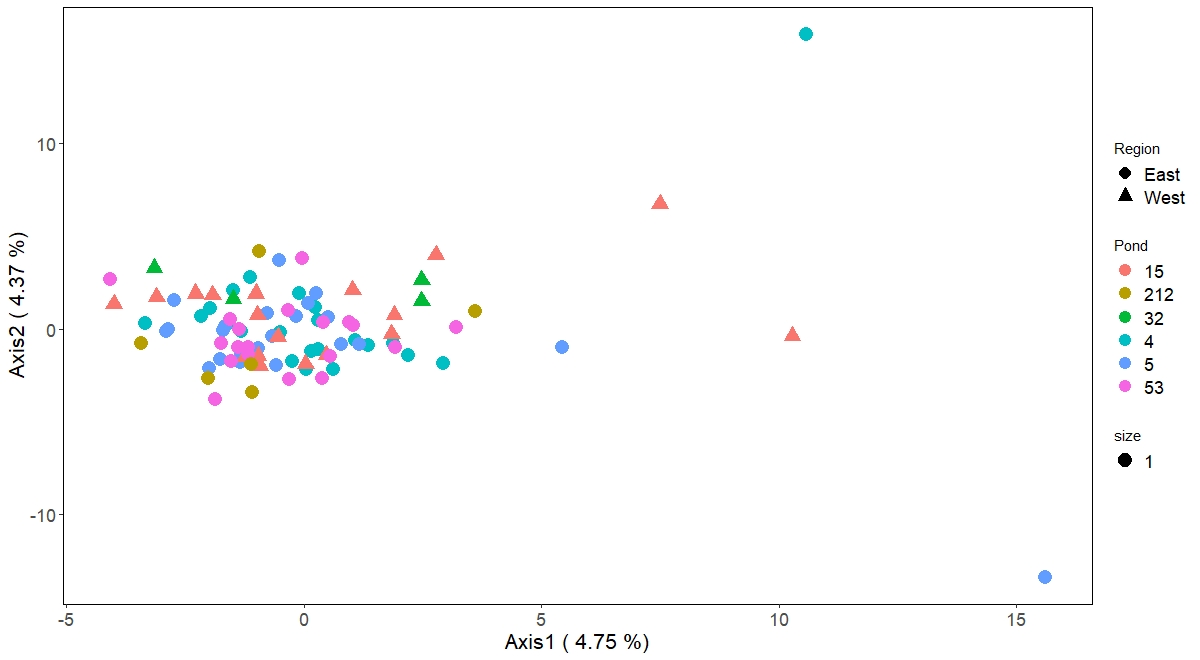


Figure S1. PCA analysis SNP markers for Eglin AFB.


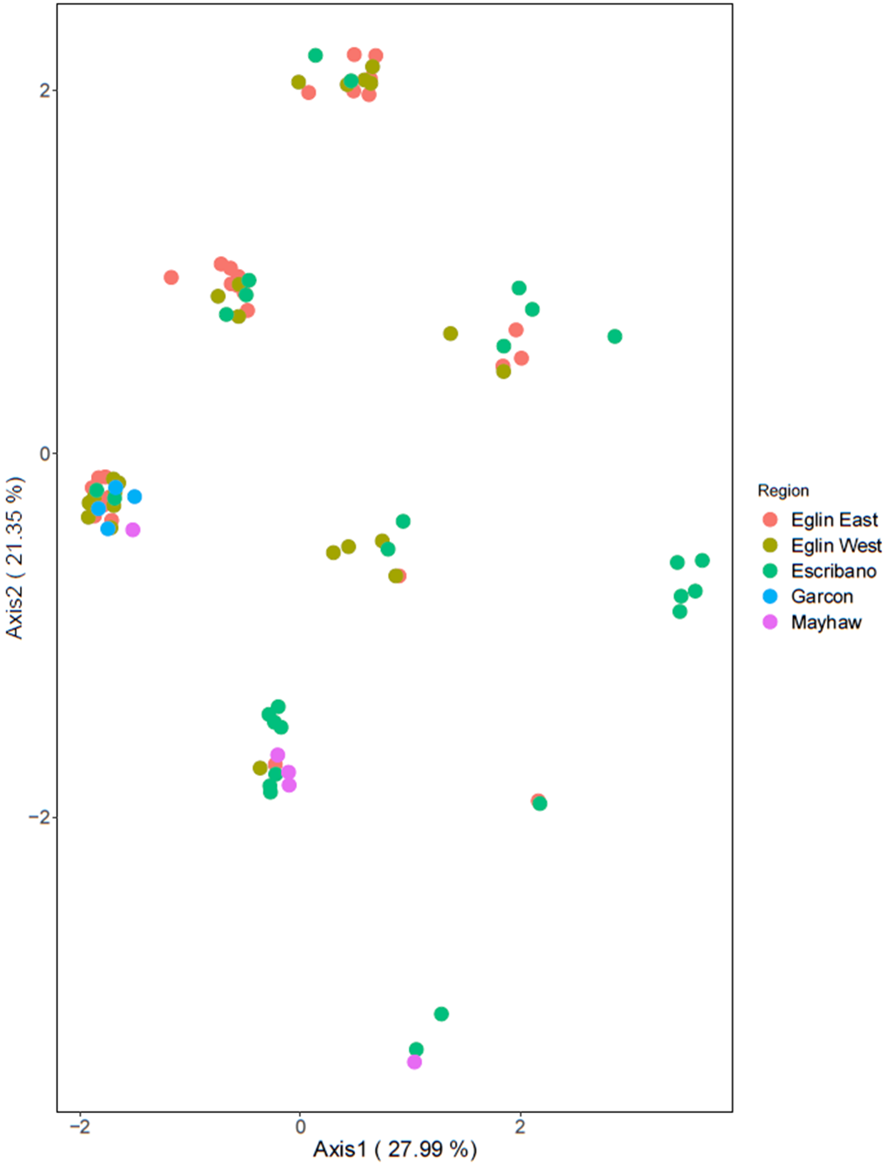


Figure S2. PCA analyses of MHC class I & II exons for all breeding sites.

Table S3. Migrate-N posterior distributions of theta (ϴ) and migration rates using SNPs, MHC class IIβ, and mtDNA.

| Marker | Parameter | 2.5  Percentile | | 25.0  Percentile | Mode | 75.0  Percentile | 97.5  Percentile | Median | Mean |
| --- | --- | --- | --- | --- | --- | --- | --- | --- | --- |
| SNP | ϴ_West Eglin_ | 0.0000 | 0.0000 | | 0.0005 | 0.0100 | 0.0250 | 0.0105 | 0.0015 |
| SNP | ϴ_East Eglin_ | 1.4380 | 1.4600 | | 1.4745 | 1.4870 | 1.5000 | 1.4725 | 1.4610 |
| SNP | East Eglin -> West Eglin | 1.7 | 6.3 | | 10.1 | 13.6 | 18.3 | 10.5 | 10.3 |
| SNP | West Eglin -> East Eglin | 1.0 | 1.0 | | 1.2 | 3.7 | 8.7 | 3.8 | 2.6 |
| MHC IIβ | ϴ_East Eglin_ | 0.0064 | 0.0089 | | 0.0108 | 0.0148 | 0.0253 | 0.0134 | 0.0144 |
| MHC IIβ | ϴ_West Eglin_ | 0.0042 | 0.0056 | | 0.0090 | 0.0138 | 0.0272 | 0.0127 | 0.0141 |
| MHC IIβ | ϴ_Escribano_ | 0.0049 | 0.0065 | | 0.0108 | 0.0169 | 0.0294 | 0.0154 | 0.0160 |
| MHC IIβ | East Eglin -> West Eglin | 1111.7 | 1241.7 | | 1315.8 | 1388.3 | 1533.3 | 1320.8 | 1321.3 |
| MHC IIβ | East Eglin -> Escribano | 678.3 | 795.0 | | 862.5 | 933.3 | 1078.3 | 872.5 | 874.5 |
| MHC IIβ | West Eglin -> East Eglin | 280.0 | 338.3 | | 372.5 | 406.7 | 471.7 | 375.8 | 375.6 |
| MHC IIβ | West Eglin -> Escribano | 1030.0 | 1176.7 | | 1260.8 | 1345.0 | 1511.7 | 1267.5 | 1269.7 |
| MHC IIβ | Escribano -> East Eglin | 701.7 | 785.0 | | 832.5 | 880.0 | 973.3 | 837.5 | 837.4 |
| MHC IIβ | Escribano -> West Eglin | 60.0 | 105.0 | | 132.5 | 160.0 | 213.3 | 137.5 | 136.8 |
| mtDNA | ϴ_East Eglin_ | 0.0004 | 0.0007 | | 0.0010 | 0.0013 | 0.0020 | 0.0011 | 0.0012 |
| mtDNA | ϴ_West Eglin_ | 0.0001 | 0.0002 | | 0.0003 | 0.0005 | 0.0009 | 0.0004 | 0.0004 |
| mtDNA | ϴ_Escribano_ | 0.0001 | 0.0003 | | 0.0005 | 0.0007 | 0.0012 | 0.0006 | 0.0006 |
| mtDNA | East Eglin -> West Eglin | 0.0 | 10.0 | | 271.0 | 1008.0 | 2624.0 | 997.0 | 1135.9 |
| mtDNA | East Eglin -> Escribano | 0.0 | 0.0 | | 237.0 | 998.0 | 2646.0 | 999.0 | 1140.3 |
| mtDNA | West Eglin -> East Eglin | 0.0 | 0.0 | | 63.0 | 396.0 | 1614.0 | 397.0 | 549.3 |
| mtDNA | West Eglin -> Escribano | 0.0 | 0.0 | | 77.0 | 714.0 | 2422.0 | 715.0 | 908.3 |
| mtDNA | Escribano -> East Eglin | 4.0 | 326.0 | | 759.0 | 1394.0 | 2630.0 | 1167.0 | 1258.0 |
| mtDNA | Escribano -> West Eglin | 84.0 | 524.0 | | 921.0 | 1734.0 | 2824.0 | 1355.0 | 1411.2 |

Figure S3. Distruct plots for SNP data organized by pond.


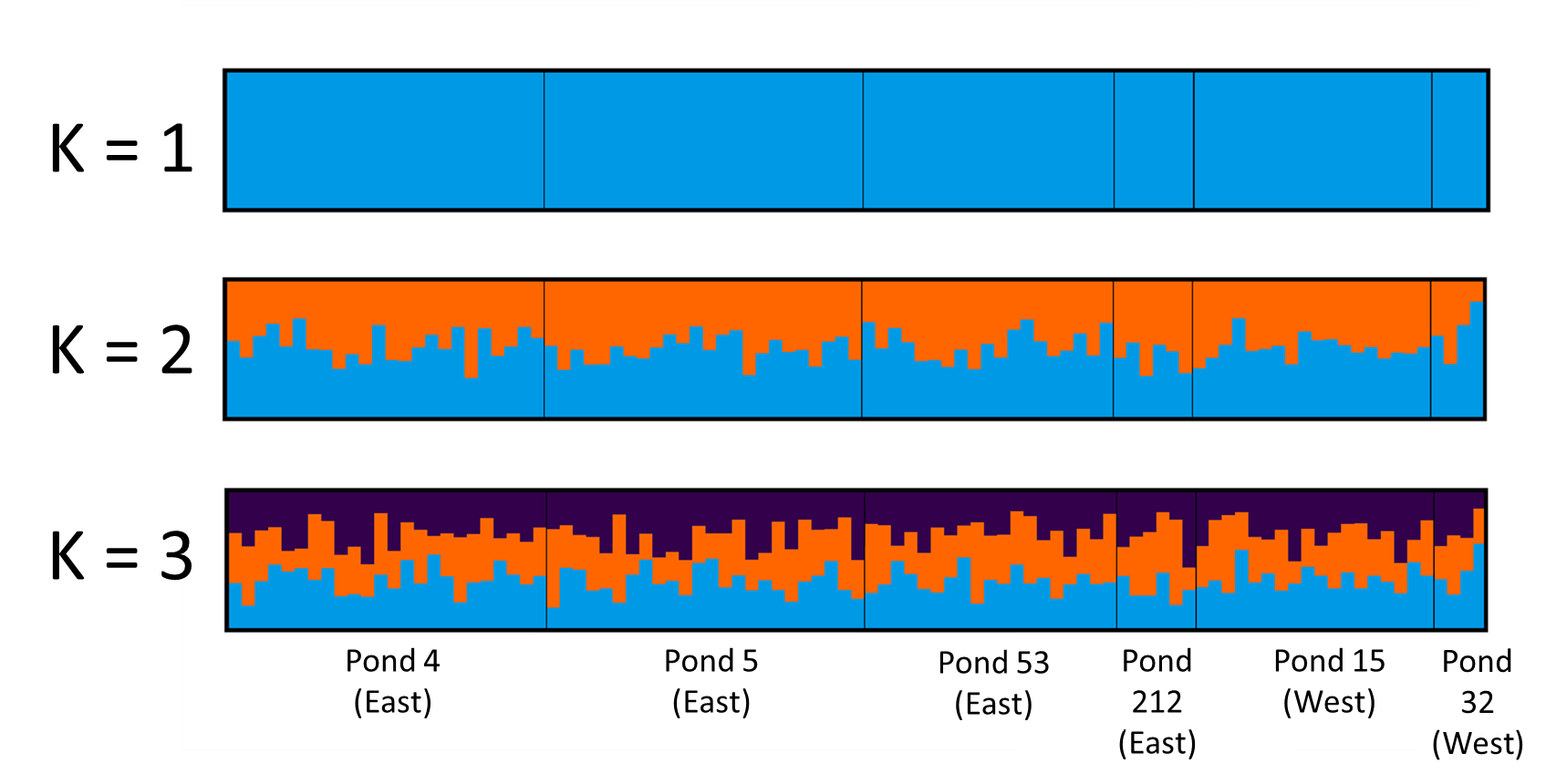


Table S4. BayesAss immigration rate estimates with 95% credible intervals using MHC and SNP datasets.

| Marker | Source | Destination | Migration Rate | 95% credible interval |
| --- | --- | --- | --- | --- |
| MHC | Eglin East | Eglin West | 0.1542 | 0.0818 - 0.2266 |
| MHC | Eglin East | Escribano | 0.0560 | 0.0172 - 0.0948 |
|  |  |  |  |  |
| MHC | Eglin West | Eglin East | 0.0964 | 0.0398 - 0.1362 |
| MHC | Eglin West | Escribano | 0.0756 | 0.0298 - 0.1214 |
|  |  |  |  |  |
| MHC | Escribano | Eglin East | 0.0623 | 0.0240 - 0.1006 |
| MHC | Escribano | Eglin West | 0.1437 | 0.0743 - 0.2131 |
|  |  |  |  |  |
| SNP | East Eglin | West Eglin | 0.3201 | 0.3072 - 0.3330 |
| SNP | West Eglin | East Eglin | 0.0056 | 0.0000 - 0.0114 |


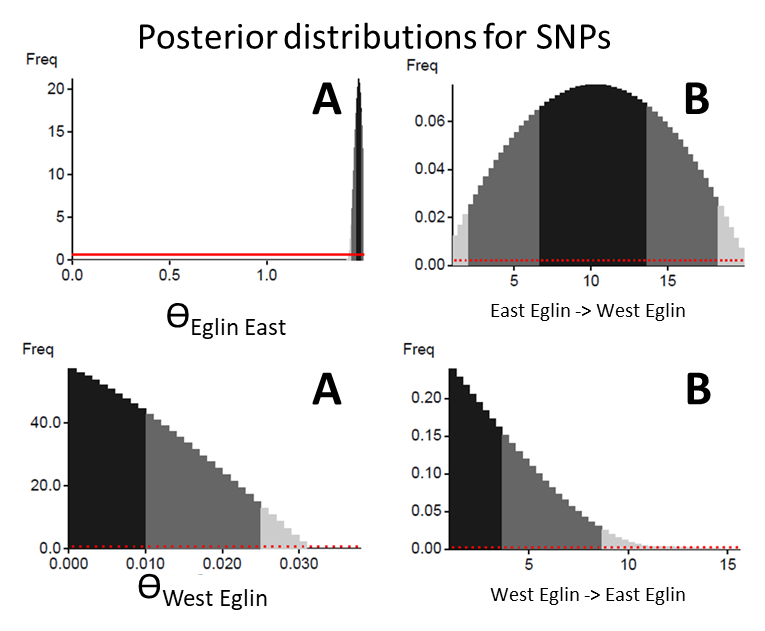


Figure S4. Migrate-N posterior distribution of theta (ϴ) (A), and mutation scaled migration rate per generation (B) using SNP data. The red lines are prior distributions used in the model.


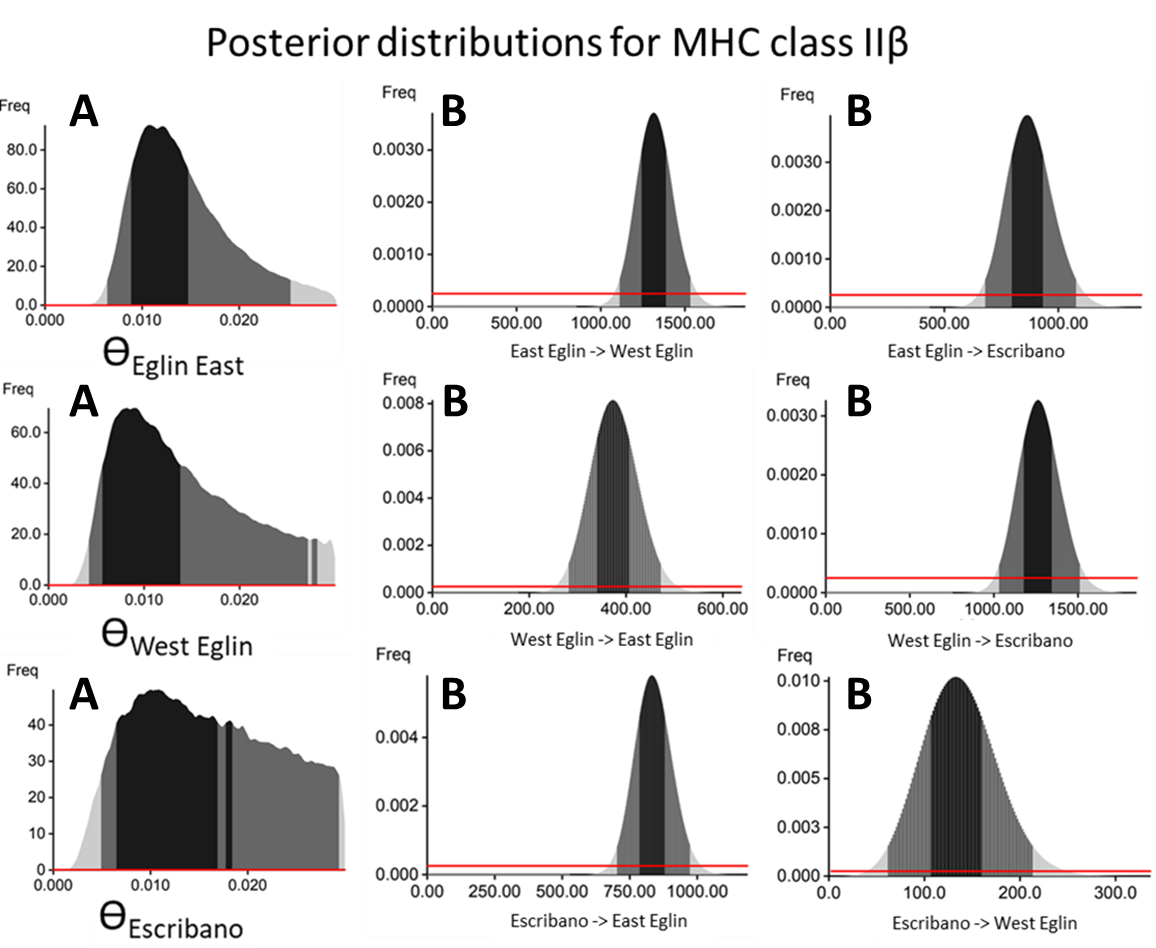


Figure S5. Migrate-N posterior distribution of theta (ϴ) (A), and mutation scaled migration rate per generation (B) using MHC class IIβ. The red lines are prior distributions used in the model.


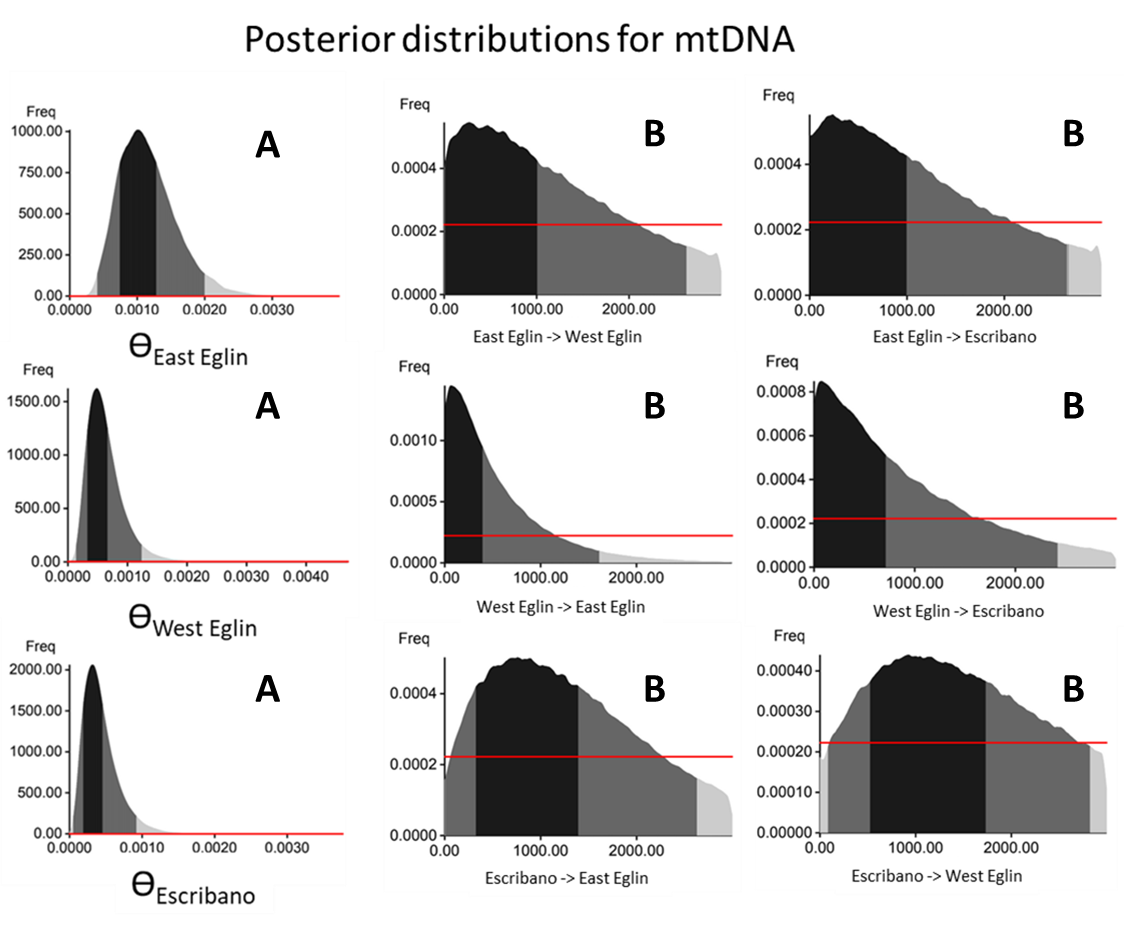
 Figure S6. Migrate-N posterior distribution of theta (ϴ) (A), and mutation scaled migration rate per generation (B) using mitochondrial DNA. The red lines are prior distributions used in the model.
